# The Future of Medical Tourism for Individuals’ Health and Well-Being: A Case Study of the Relationship Improvement between the UAE (United Arab Emirates) and South Korea

**DOI:** 10.3390/ijerph19095735

**Published:** 2022-05-08

**Authors:** Haeok Liz Kim, Sunghyup Sean Hyun

**Affiliations:** 1 Computational Social Science Center, Hanyang University, 222, Wangsimni-ro, Seongdong-gu, Seoul 04763, Korea; lette0704@hanyang.ac.kr; 2 School of Tourism, Hanyang University, 222, Wangsimni-ro, Seongdong-gu, Seoul 04763, Korea

**Keywords:** individuals’ health, medical tourism, medical treatment, international politics, South Korea, United Arab Emirates

## Abstract

The medical tourism industry has been growing rapidly in recent years as governments seek new sectors for sustainable growth. The increase in medical tourism and cooperation in the health and medical sector may be a path to improving relationships between countries. As a result of globalization, international tourism has been growing faster than any other time in the past. The growth of international tourism is being regarded as a contributor to the development of the economy, social cooperation, politics, and cultural relations. This paper suggests that developing medical tourism may act as a basis for increasing tourism in general, thereby enhancing cultural exchanges and improving the relationship between South Korea (Republic of Korea) and the United Arab Emirates (UAE), especially by establishing a new cooperative relationship in the health and medical field. This paper focuses on the functions of medical tourism in the past and its potential in the future, which may play a significant role in the relationship between South Korea and the UAE, particularly its influence on South Korea’s policies of cooperation with the UAE in the field of health and medical services.

## 1. Introduction

As life expectancy grows, so too does people’s interest in individual health and quality of life. Furthermore, as travel has been facilitated by globalization, the demand for medical tourism to take care of individual health has also increased. Cross-border travel for healthcare, the ease of travel between countries, and increased health awareness and healthcare expectations have contributed to making medical tourism a growing industry in many countries [1]. In 2020, the largest medical tourism market in the US was estimated at US $18 billion, and this figure is projected to reach US $31.2 billion by 2027 [2]. The international medical tourism market is expected to grow by USD 131.35 billion by 2025, with an average annual growth rate of 20% [3]. Medical tourism is an important part of a steady state [4]. The United Arab Emirates (UAE) has taken steps to increase its medical tourism because tourism is an economic pillar [5]. In 2019 (until the outbreak of the COVID-19 pandemic), Dubai became the world’s fourth-largest tourist destination city. UAE cities, such as Dubai and Abu Dhabi, have become some of the most developed cities in terms of tourism, attracting many tourists for medical tourism as well [5].

However, the COVID-19 pandemic has affected the socio-economic and political stability of countries. In 2020, the World Travel and Tourism Council (WTTC) (2021) estimated that about 62 million jobs would be lost (18.5%) worldwide, and the travel and tourism industry’s GDP contribution would be decreased by 49.1% [6,7]. This, of course, impacts the medical tourism sector as well. For example, the number of foreign patients in South Korea has decreased in 2020 compared to 2019 due to the COVID 19 pandemic [8]. The outbreak of the COVID-19 pandemic played a role as a restraint on the growth of the medical tourism market between 2020 and 2021.

Prior to the spreading of the coronavirus, medical tourism was an important growth industry in many emerging economies. Although the medical tourism market is currently stagnant due to the coronavirus, this study assumes the medical tourism market after the coronavirus pandemic comes to an end.

The growth in medical tourism is largely due to the decreasing costs of travel and the increase in advertising by companies that wish to attract patients, as well as the improved availability of health technology [9]. Although this change has mainly been led by individuals, medical tourism has also received institutional endorsements [1]. Over the past decade, tourism authorities and community leaders have begun to consider medical tourism a significant industry with the potential to diversify existing forms of tourism, enhance local health care systems, improve the economy, increase tax revenues, and create employment [10]. Medical tourism originally emerged from “health tourism,” which involved traveling outside one’s natural health care jurisdiction to restore individual health and enhance medical treatments and wellness [10,11]. Medical tourism is market-driven: it is shaped by the complicated interactions of a myriad of economic, medical, social, and political forces [12].

The political and economic situations in Middle Eastern countries have a significant influence on South Korea since it is dependent on the region for energy (more than 90% of its energy imports come from the Middle East). Therefore, instability in the Middle East directly affects South Korea’s national security, as well as the safety of Korean citizens there [13]. This implies that South Korea must establish a more comprehensive and interdependent relationship with the Middle East. To this end, South Korea’s government has built a durable partnership based on shared visions and goals to go beyond the existing areas of cooperation in energy construction and set out for collaboration in new areas, such as IT, nuclear and renewable energy, and health and defense [13].

Although Middle Eastern patients account for a small portion of the medical tourists visiting South Korea, the rate of patients coming from the UAE is increasing at a relatively high rate. Patients that went to South Korea from the UAE increased from 158 in 2011 to 2633 in 2014, while the revenue generated from UAE medical tourism increased from 20.4 billion won (19.5 million USD) in 2013 to 40.5 billion won (37.2 million USD) in 2014 [14]. This is significant in that, among patients from the UAE, the medical service revenue earned from an individual patient per capita is the highest of all countries; it reached 15.4 million won (12.7 thousand USD) in 2014, which is more than 7.4 times higher than the total average of 1.9 million won (1.6 thousand USD) [14].

Among all the Middle Eastern countries, the UAE has emerged as South Korea’s most important partner. In the process of strengthening the diplomatic relationship between the two countries, cooperation in the traditional construction and energy industries was expanded to include the private sector. This cooperation now includes the field of medical tourism, which is now recognized as a new engine for growth.

Along with the increase in medical tourism, the governments of the two countries started seeking more cooperation in the health and medical fields. As well as being the Middle Eastern country with the most medical cooperation with South Korea, the UAE was also the first to form a comprehensive strategic partnership with the state (i.e., when South Korea won its first nuclear power plant contract in 2009) [14]. In March 2015, when former President Geun-hye Park visited the UAE for the second time, she emphasized the need to expand cooperation in the field of health and medical services, along with some other areas that are considered high-valued soft industries [15].

This paper focuses on the functions of medical tourism in the past and its potential in the future, which is expected to play an important role in the international relations between South Korea and the UAE. This paper argues that developing medical tourism and attracting patients from the UAE have played a role in improving South Korea’s relationship with the UAE. This has influenced the government’s policies of cooperation with the UAE in the field of health and medical service.

Additionally, a major characteristic of tourism is that it naturally increases the interaction between the citizens of the host and guest countries, thereby having the potential to foster cultural exchanges and create a better understanding between the citizens of each country. This, in turn, may positively contribute to the diplomatic relations between the two states. This paper argues that developing medical tourism may be an important factor contributing to the increase in tourism in general, enhancing cultural exchange, and, as a result, improving the relationship between South Korea and the UAE. This paper begins by analyzing the existing research on the political aspects of tourism.

Hall (1994) illustrates a comprehensive relationship between tourism and politics while also pointing out that “despite the vast amount of research currently being conducted elsewhere in the social sciences on tourism-related subjects, the politics of tourism is still the poor cousin of both tourism research and political science and policy studies” [16]. This paper focuses on the aspects of research related to international tourism policy and international relations as a basis for the argument that tourism may influence international relations.

Butler and Suntikul (2017) describe the relationship between politics and tourism in the following way: “new political constellations emerge with the shifting economic fortunes and political affiliations of countries around the world, and xenophobic religious fanaticism ascends to the global political stage” [17]. Burns and Novelli (2006) focus on the complexities between tourism and the pursuit of social and economic harmony. Burns’s perspective of tourism supports and highlights the importance of cultural factors in South Korea and the UAE’s medical tourism. The mix of politics, culture, and social identities has raised some important issues [18]. Urry (2002) uses the term “liquid modernity” coined by Zygmunt Bauman to explain the close relationship between globalization and tourism in the future [19]. As the book only hypothesizes on the theory between the two relationships without providing any hard evidence [19], this paper uses the theory but supports it with other studies with a more scholarly perspective.

Based on the previously cited literature, this paper also argues that international tourism is essentially related to international politics and illustrates the aspects of this relationship. In terms of globalization, this research argues that international tourism may have a positive influence on the cooperation between the governments.

As such, this paper looks into the relations of South Korea and the UAE from the perspective of political, economic, and cultural cooperation. Available governmental research and other reference materials have been used, including publications from South Korea’s Ministry of Foreign Affairs, to assess the relations between the two countries. Furthermore, the paper focuses on the relations between these two countries from the perspective of the increase in medical tourism. To support this research, the materials published by South Korea’s Ministry of Health and Welfare and other government-affiliated institutes, such as the Korea Health Industry Development Institute, have been used for reference. Based on these materials, this paper concludes that the medical tourism from the UAE to South Korea has increased at a significant rate.

One of the major factors that encourage South Korea to seek comprehensive cooperation relationships with other countries is the fact that South Korea is highly dependent on other countries for energy. Keohane and Nye (2012) clearly illustrate theories of interdependency; their basic argument is that “Interdependence and globalism raise political issues: individuals and groups continue to struggle for advantage and to turn the tides of globalism in their favors” [20]. As globalization proceeds, both political cooperation and economic interdependence become increasingly important.

The medical tourism industry has been growing rapidly as governments have been seeking new sectors for sustainable growth. The increase in medical tourism and cooperation in the health and medical sectors may be a path to improving relations between countries. In other words, medical tourism can be treated as a variable in the relations between the two countries. Therefore, this paper focuses on the relationship between South Korea and the UAE among other Middle Eastern countries. The UAE has been chosen because it has the highest number of medical tourists that have visited South Korea, and it has also established the most health and medical service agreements. As such, this case study is expected to shed some light on how South Korea’s relationships with other countries in the region may develop in the future.

## 2. Literature Review

### 2.1. Theoretical Perspectives of Tourism in International Politics

This paper addresses the political and economic dimensions of international tourism and seeks to answer the question of whether international tourism has a positive influence on international relations. In March of 2015, former Minister of Health and Welfare Moon Hyung-pyo said, “The UAE is the first Middle Eastern country to form a comprehensive strategic partnership with Korea in 2009 when Korea won its first nuclear power plant contract and at the same time it is the No. 1 Middle Eastern country in the area of medical cooperation with Korea” [21].

Richter (2013) poses an array of questions on the relations between international tourism and international relations: “Under what circumstance can tourism promote reconciliation among nations? Why is it so often the earliest component of a normalization process between two nations? To what extent does becoming a host nation improve national understanding of other societies? Under what conditions does tourism intensify hostility, friction, and feelings of relative deprivation? How and by what process do travelers’ views become politically important?” [22].

International tourism has usually been assessed from the economic or commercial perspectives, with a primary focus on the economic impact it has on the national industry. However, by nature, international tourism is inseparable from the field of international relations. The extent of tourism between two nations reflects the political relations between them. Tourism is sometimes also used as a means of improving a nation’s international political image. For example, Hall (1994) has shown how the regime of former President of the Philippines Ferdinand Marcos launched a vigorous tourism development program to maintain and justify the Marcos dictatorship with foreign governments and investors [10]. The issue of interdependence in traditional international politics has implications on international tourism. Although flows of tourism between nations may encourage economic interdependence, there is criticism that the uneven flow of tourists from developed countries to Third World regions creates economic dependence [16].

An aspect of tourism in international relations that many find attractive is that tourism can function as a “force for peace,” or, as the United Nations World Tourism Organization’s 1967 slogan goes, as a “passport for peace” [23]. Based on this notion, the 1975 Helsinki Accord encouraged the development of tourism as a means of achieving security and cooperation in Europe [16]. Therefore, scholars must consider the time that has passed since the 1975 Helsinki Accord. Since then, globalization has had an impact on international society and, as a result, international tourism has increased rapidly. According to the World Bank’s “International tourism, number of arrivals” data, in 2019, around 2.4 billion tourists travelled internationally, a 42.0% increase compared to 2009 (1.69 billion) [24]. As such, international society has placed more emphasis on using tourism as a means of peace.

The increase in international tourism impacted by globalization may have negative effects on peace and the improvement of international relations. In *Tourism and Political Change*, Suntikul and Butler argue that the 2002 bombings of tourist nightlife venues in Bali, and the use of commercial jets to destroy New York’s World Trade Center in 2001, are indicative of the tourism industry’s attractiveness and vulnerability as a target for politically motivated terrorist attacks [17].

This paper focuses on the positive effects of tourism on current international relations. Although it is still contested whether it is “tourism—passport for peace” or “peace—passport for tourism,” tourism is essential in fostering a path for improving relationships and increasing cooperation between nations.

### 2.2. The Growing Importance of Medical Tourism in International Relations

Medical tourism is primarily a late twentieth-century phenomenon, which is said to be rapidly expanding. Medical tourists are now traveling abroad to improve their health instead of relying solely on their nations’ medical service. Medical tourism has been generalized as something of an umbrella term associated with travelers in search of better health, which includes not only wellness therapies bundled with services for food and beverage, lodging, entertainment, and touring or exploring the attractions of a destination but also elective and necessary medical (surgical and nonsurgical) procedures [10,11,25].

According to an OECD ((Organization for Economic Cooperation and Development) report “Medical Tourism: Treatments, Market and Health system Implications: A scoping review,” medical tourism is defined as consumers electing “to travel across international borders with the intention of receiving some form of medical treatment” [26]. This treatment may span the full range. Similarly, Hall (2011) defines medical tourism as “the main motive of the tourism concept in tourism services that are related to health and medical tourism section” [25].

Numerous factors have influenced the spread of medical tourism. One basic factor is that people are now more interested in health than ever. The UN Millennium Poll of 2000, a global survey commissioned by the United Nations, consistently ranked health as people’s number one desire [27]. Moreover, health and well-being are being recognized as an important basic human right. For example, Article 25 of the Universal Declaration of Human Rights states that “Everyone has the right to a standard of living adequate for the health and well-being of himself and of his family. It is a fundamental human desire” [27].

Globalization is a major contributing element to the expansion of medical tourism. According to the McKinsey Report 2008, a typical phenomenon of medical tourism, the objective of global convergence in medical tourism, has generated the demand for the most advanced medical technology and better service [28]. There are a variety of reasons why people prefer medical tourism over domestic medical services. Seeking the most advanced medical technology (40%) is the main motivator for medical tourists, followed by better essential medical treatment services (32%) [28]. Other reasons include quicker access to medical services (15%) and lower costs (9%) [28]. These diverse purposes of medical tourism are being analyzed by policymakers in order to strengthen competitiveness.

As the medical tourism market is growing more profitable, governments are striving to secure a strong position for their medical sectors. For example, Singapore’s government has planned to become the medical hub of Asia and established “Singapore Medicine,” an institute that seeks to promote Singapore as a destination for medical tourism. Thailand has also launched the “Medical Hub of Asia Project” and has developed a variety of tourism packages [29]. South Korea’s government has designated medical tourism as a new growth engine industry, and government-affiliated institutes such as the Korea Health Industry Development Institute (KHIDI) are developing policies to promote medical tourism.

## 3. Materials and Methods

This paper argues that medical tourism may act as an important factor in the improvement and enhancement of the relationship between South Korea and the UAE. In this regard, this paper will attempt to answer the following questions:

1. Is there a relationship between tourism and international politics, and can the promotion of tourism improve relations between the two countries?
2. Has medical tourism between South Korea and the UAE worked to improve the relationship between the two countries, and, if so, in what respect?

In terms of an answer to the first question, tourism and international politics are not separate issues. Instead, they are correlated in many ways. As globalization has an impact on the development of tourism in many ways, tourism between countries influences their political and diplomatic relations. This paper assumes that tourism plays a vital role in international politics.

In terms of the second question, South Korea has established relations with the Middle East, although this relationship is mostly one way as South Korea is dependent upon those countries. In interdependency theory, this is called vulnerability, and a good way to supplement such a relationship is by establishing a comprehensive and cooperative relationship. Establishing a new field of cooperation through the promotion of medical tourism is a good way to establish this. Moreover, despite the political and economic importance of Middle Eastern countries to South Korea, cultural exchanges with them remain marginalized. The promotion of medical tourism may play a role in enhancing cultural exchanges between them.

**Figure 1:**
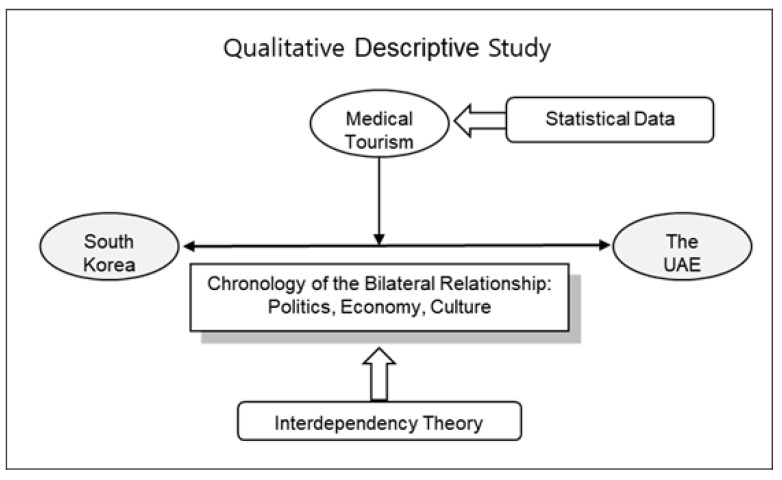
Flowchart of the research methodology.

Figure 1 shows the flowchart of the research methodology based on qualitative descriptive study design. To review the implications of establishing cooperation in the health and medical fields between South Korea and the UAE, this paper refers to a report published by the Korea Institute for International Economic Policy, titled “The Health Care Industry in the MENA (Middle East and North Africa) Region and Its Policy Implications for Korean Companies” [30]. This report analyzes the health and medical industry of countries in the MENA region, medical tourism activities, the countries’ policy plan of potential developments in the field, and analyzes the cooperative and collaborative environment for possible cooperation along with policy suggestions [30]. Then, this paper briefly looks into the bilateral relationship between South Korea and the UAE in terms of politics, economics, and culture, and then analyzes its aspects of medical tourism. This paper then suggests the implications of medical tourism between South Korea and the UAE. The implication here is that medical tourism may function as a medium for health and medical cooperation—a broader political aspect—between the two countries, and as a factor in expansion of cultural exchanges. This paper concludes that medical tourism may not just act as a simple aspect of tourism or development of a new industry but as a path to improve the relationship.

## 4. Results

### 4.1. The Bilateral Relationship between South Korea and the UAE

#### 4.1.1. Politics

The relations between South Korea and the Middle East have not received much attention as South Korea’s diplomatic relations have previously been more focused on the U.S., Japan, and China. As mentioned in “The Republic of Korea and the Middle East: Economics, Diplomacy, and Security” by Levkowitz (2010) [31], “South Korean–Middle Eastern relations have been neglected in the literature throughout the years, mainly owing to the focus on Korea’s relations with the United States and Asian states and the attention given to the North Korea.” Since the mid-1970s, South Korea has gradually recognized the importance of the relationships with Middle Eastern countries based on economic cooperation [31]. The diplomatic relations between the two countries were established in 1980 for economic reasons. Table 1 shows their chronology [32].

However, it was only in the 2000s that the diplomatic relations started to develop faster. Table 2 shows the major agreements between South Korea and the UAE.

In recent years, the diplomatic relations between South Korea and the UAE have been developing even faster. In December 2009, when a South Korea consortium led by Korea Electronic Power Cooperation won the contract to construct the Braka nuclear power plant (the largest scale of South Korea’s overseas construction contracts ever signed), the UAE became South Korea’s only strategic partner in the Middle East [32,33]. Additionally, the UAE was the first country that President Geun-hye Park visited twice, following visits to the US and China. The UAE has also made official announcements addressing North Korea’s provocation [34]. Cooperation in the field of national defense has also been developing. In 2011, South Korea started deploying military forces to help train the UAE’s Special Forces, and cooperation is also anticipated in the field of anti-cyber terrorism [34]. At the level of ministers and high-level officials, many visits have taken place, the last of which was the official visit of His Highness Sheikh Abdullah bin Zayed Al Nahyan, Minister of Foreign Affairs and International Cooperation, to Seoul in July 2020 [32].

#### 4.1.2. Economy

According to the Korea International Trade Association, the UAE is South Korea’s twentieth-largest export country and tenth-largest import country. In 2014, the trade volume amounted to $23.4 billion. In 2013, South Korea’s exports dropped to $5.7 billion from $6.9 billion the previous year but bounced back to $7.2 billion in 2014.

The imports were relatively higher than the exports, amounting to over $15 billion for each of the last three years. Although the increase rate of exports was higher than the increase rate of imports, the import amount was still 2.3 times higher in 2014 [35]. The major export items from South Korea to the UAE include automobiles, mobile phones, automobile components, steel structures, and color television sets [29]. The amount of exports decreased by $5.5 billion over the last five years. South Korea’s major import items from the UAE are primarily comprised of energy, such as crude oil, petroleum, and LPG (liquefied petroleum gas) [35].

In 2020, the volume of the UAE’s exports to South Korea reached $ 5.693 billion while the volume of exports from South Korea to the UAE reached $ 3.694 billion [32]. Crude oil is the most important export of the UAE to South Korea (about 90% of total exports). The most important Korean exports to the UAE are technological products, such as cars, telephones, ships, electrical tools, and display screens. The total amount of Korean investments in the UAE reached US$ 1.4 billion compared to US$ 0.45 billion for UAE investments in South Korea.

#### 4.1.3. Culture

Recently, there has been an increase in activities that aim to foster a better understanding between the two cultures through various cultural and educational exchanges. South Korea has been developing public diplomacy, such as expansion of exchanges between universities and introducing performing arts in the UAE. This should be understood as an attempt to expand the political and economic cooperation into more comprehensive fields. To promote the cultural exchanges between the UAE and South Korea, the two sides signed the cultural cooperation agreement in 2007. In line with that, exchange of cultural events took place in both countries.

In June 2013, a UAE Youth Ambassador Program was held by the Abu Dhabi government. Additionally, a “Korea Week 2013 in UAE” festival was held, and the first Korean speech contest took place in November of the same year. Universities from the two countries have been cooperating significantly over recent years. Furthermore, in 2015, a Korea Festival was held, as well as the first Korea–UAE Taekwondo tournament. A Korea information room was also opened in the Abu Dhabi National Library and a few K-pop concerts were held. These recent events may not seem to be many, but they have substantially increased in comparison to the past, which reflects the expanding cultural exchanges between the two nations.

In March 2015, a Memorandum of Understanding was signed by the Foreign Ministries of South Korea and the UAE on the Establishment of a Korean Cultural Center in the UAE for the Promotion of Cultural Cooperation. It was the first establishment of a Korean Cultural Center in a member country of the Gulf Cooperation Council (GCC) [33].

To celebrate the 40th anniversary of the diplomatic ties between the two countries, the culture ministers of the two countries announced 2020 as the Year of Cultural Dialogue. The event has been extended to 2021 amid the current novel coronavirus (COVID-19) outbreak, which has disrupted various cultural events [32].

### 4.2. The Contemporary Status of Medical Tourism and the Bilateral Relationship between South Korea and the UAE

#### 4.2.1. The Middle Eastern Countries’ Medical Tourism to South Korea

Middle Eastern countries’ interest in South Korea is understandable. South Korea’s medical tourism links popular treatment efficiency with professionalism in terms of high levels of medical technology. The medical tourists in South Korea may expect the best medical services in the world. South Korea has been steadily advancing in the industry of medical tourism. There are many reasons as to why South Korea promotes medical tourism, as explained in the research on the rise of foreign medical tourists since 2009. According to the article on “Sustaining Medical Tourism in South Korea” mentioned by the US–Korea Institute at the Johns Hopkins School of Advanced International Studies (SAIS), “Korea exploits rising health care costs and increased waiting times in the United States to its advantage. Compared to the United States, Korea’s prices are inexpensive and waiting times are short. Furthermore, legislation encourages medical personnel exchanges, particularly with the United States. Collaboration, training, and contact with the U.S. health care system attracts additional American patients” [36].

First, South Korea has benefits that include low costs of medical treatment compared to other medical tourism destinations. Despite South Korea’s high level of experience and expertise, the medical care costs are considerably lower than in the US or Japan. Table 3 illustrates the difference in medical costs between South Korea and the US. In 2010, the surgery costs for some diseases were about five times cheaper than in the US. The hospitals in South Korea offer excellent medical services at low costs in order to attract foreign patients.

Second, accessibility is one of the strengths of South Korea’s medical tourism. Medical tourists have easy access to information regarding it. Additionally, South Korea is well-known for its registration system, which is an attractive feature for foreign patients. The Ministry of Health and Welfare has started enforcing the registration system in 2009 for the institutions serving foreign patients in Korea, making way for the development of the mandatory medical malpractice liability insurance in 2016. All of these aspects were established to secure safer and higher-quality Korean medical services for foreign patients. Handley (2015) [36] states that medical procedures in South Korea present advantages for medical tourism. South Korea is famous for specialized treatment, attracting patients with its exceptional treatment, and also continues to develop specialization facilities and medical personnel.

Third, the advancement of technology constitutes a favorable reason for South Korea’s medical tourism. The article about sustaining medical tourism in South Korea stressed that “biotechnology was one of the original driving forces behind the Korean medical tourism impetus; it is not surprising that Korea’s biotechnology is state of the art” [36].

Remarkable skills in that area result in attracting patients that need short-term visits. In 2009, which was a year of the market-research survey related to international patients visiting Korea for medical tourism, 48% of the patients cited “the quality of medical service and technology as their central reason for choosing Korea” [2].

The total number of foreign patients has increased four times since 2009 with a rapid average increase rate of 23.5% per year. The total number of medical tourists that visited Korea in 2019 amounted to 497,464 patients, which represented a 31.3% increase compared to the previous year. Table 4 shows the total number of foreign patients based on nationality [14].

Table 4 illustrates that the number of foreign patients has increased steadily at an average YoY (year on year) rate of 18.59%. The largest increase rates were in patients from Kazakhstan, then Vietnam, followed by those from the UAE. The Ministry of Health and Welfare stated that the increase in patients from the UAE (59.6%) was a result of the government patient transfer arrangements signed with the Health Administration of Abu Dhabi in 2011 and with integrated groups of the UAE in 2013 [37].

Medical tourists from Middle Eastern countries tend to prefer Western countries, such as Britain, Germany, and the US, for medical treatment. Before 1997, the US and Europe were the centers of the healthcare industry. Yet, as medical tourism is a form of tourism, it is also affected by the political events that affect tourism. A political factor that changed the trends of medical tourism among Middle Eastern citizens was the 9/11 terror attack, which had a significant impact on tourism in the US. According to the paper “The Terrorist Attacks of 9/11 and the Financial Crisis of 2008: The Impact of External Shocks on U.S. Hotel Performance,” the “U.S. hotel industry faced two major external shocks in the decade of the 2000s, the terrorist attacks of 11 September, 2001, and the financial crisis of September 2008, which led to an economic recession” [38].

Following this point, the industry underwent a great change worldwide. Since 9/11, the US has tightened its security and immigration procedures. Furthermore, since anti-Islamic sentiments increased after 9/11, the Middle East’s medical tourists started to turn their attention to Asian countries, including Thailand, Singapore, India, and South Korea. As such, these Asian countries experienced many benefits after this point due to the increase in medical tourists from the Middle East.

Table 5 shows the medical treatment status of Middle Eastern patients visiting South Korea for medical tourism purposes. Integrated internal medicine (general, infectious, respiratory, allergic internal medicine, etc.) accounted for the largest proportion, and the dermatology and oriental medicine departments made up the next-largest proportions.

#### 4.2.2. Medical Tourism from the UAE to South Korea

According to the KHIDI, “The demand for closer medical cooperation between South Korea and the Middle East is rapidly increasing” [14]. South Korea successfully held events in Qatar and the UAE aimed at promoting medical tourism in the Middle East. Being aware of the Middle East’s growing medical tourism market, South Korea’s government has promoted many activities to encourage Middle Eastern patients to travel there for medical purposes.

According to the statistics on the KHIDI, a total of 8963 Middle Eastern patients accounted for 1.8% of the total 497,464 foreign patients in 2019; among them, patients from the UAE accounted for the highest, with 4089 patients (45.6%). The number of patients from the UAE that visited South Korea for medical tourism was 1151 in 2013, 2633 in 2015, 3562 in 2016, 3384 in 2017, 3304 in 2018, and 4089 in 2019. The average increase rate from 2009 to 2019 was 73.0%, which is much larger than the total average of 23.5%. Although the 4089 tourists only accounted for 0.8% of all medical tourists, the increase rate is relatively high.

In March 2010, the Korea Tourist Organization (KTO) made an agreement with the Middle East’s biggest media group, Rotana Media Service, to promote South Korea’s medical tourism brand and promote tourists’ products customized for the Middle Eastern population. The two parties also agreed on utilizing entertainment programs to promote South Korea’s medical tourism through the “Star Academy Project” [39]. When the “Visit medical Korea” website was established, an Arabic language option was included. In October 2014, the KTO held a “Korea Medical Tourism Festival” at the Abu Dhabi National Exhibition Center in the UAE. Under the theme “Design Your Healthy Life in Korea,” 20 Korean medical-tourism-related enterprises, including 13 hospitals, participated. The festival also offered free medical consultations to manage the growing interest of UAE citizens.

Local governments in South Korea also participated in promoting the state’s medical tourism. For example, in December 2014, Incheon City and the Incheon Medical Tourism Foundation invited Aldar TV from the UAE to introduce Incheon’s medical facilities. This was aired on a TV program called “Buhajoos in Korea” and was also introduced in other media, such as the UAE newspaper *Emarat Al Youm***.**

South Korea has the highest potential to attract foreign patients, especially from the UAE. South Korea’s approach should be to focus on the satisfaction of medical tourists. It is necessary to prepare a long-term cooperation plan, including one for the local market. Attracting foreign patients from the UAE is a highly significant development, which can also benefit the nation’s economic and political development.

### 4.3. A New Field of Cooperation in an Interdependent Region

As illustrated in the section on economic relations, most of South Korea’s imports are related to energy, specifically crude oil, petroleum, and LPG. These three items accounted for 95% of South Korea’s imports from the UAE in 2014. What is more important is that imports from the UAE accounted for quite a substantial proportion of the total imports of those items. According to the Korean Energy Economic Institute (KEEI), 86.0% of the crude oil is imported by South Korea from the Middle East; in 2014, the UAE was the third-largest exporter of crude oil to South Korea, accounting for 11.6% of the total crude oil imported, and the largest exporter of LPG, accounting for 21.1% [40].

These energy items constitute strategic resources and are essential necessities, especially for a country like South Korea, which cannot obtain these items from its own territory, and which faces a constant risk of military conflict. According to Keohane and Nye’s *Power and Interdependence*, “interdependence most simply defined means mutual dependence. Interdependence in world politics refers to situations characterized by reciprocal effects among countries or among actors in different countries” [20].

From this perspective, South Korea is very sensitive to the supply of crude oil or LPG, and many efforts have been made to reduce this vulnerability [41]. As of September 2015, the Korean National Oil Corporation participated in 24 overseas oil exploration projects in 20 countries [42]. It seems that South Korea will still be dependent on oil from the Middle East for a long time. As of now, 86.0% of its crude oil is imported from the Middle East and most of its oil refinery facilities are designed for the features of Middle Eastern oil [43].

Moreover, as South Korea and the UAE’s relationship has developed into a strategic partnership, it is important to establish comprehensive cooperation across various fields. South Korea’s recent increase in diplomatic relations with the UAE seems to be an attempt to establish a more comprehensive relationship. For this to succeed, the interests of both nations must be met. Therefore, a good approach is to find a field that covers a wide range of governmental and private activities and establish a cooperative relationship in that field.

In this sense, the health and medical sector seems to be an adequate field. The UAE has designated the development of the health and medical sector as a national strategic task. For example, the UAE government’s 2011–2013 Strategy has designated world-class healthcare as its third priority. The promotion of medical tourism to attract patients from the UAE to South Korea may contribute to easier and more efficient government cooperation in a broader field of the health and medical sector.

Despite frequent politics and economic exchanges, the cultural exchanges are much smaller. There is not enough systematic training for the understanding of each other’s culture due to insufficient international exchanges between the two countries. Therefore, education in terms of building awareness of cultural aspects between South Korea and Middle Eastern countries ought to be strengthened.

According to “The Guidelines for Muslim Tourists,” which the world is now focusing on, the satisfaction of the Muslim tourists is highly dependent on the comfort and consideration of their religious practices [44]. Thus, the early non-Islamic nations that paid attention to the Muslim tourist market furnished the necessary facilities to promote the industry. The average annual growth in the Middle East’s medical tourists has largely increased. In general, medical tourists from the Middle East prefer to go to geographically adjacent countries in Europe or culturally similar countries. The geographical proximity and cultural similarity of South Korea is lower than other countries in Europe or Southeast Asia. Therefore, South Korea has to prepare a culturally sensitive plan for medical tourists coming from the Middle East. The important cultural considerations include religion, Islamic culture, and language skills.

Tourists from the Middle East are especially worried about food products. According to a survey conducted by the KTO among the Muslim people who visited Korea, 50.6% responded that food was an inconvenience. Aware of this problem, the government and private hospitals in South Korea started providing Halal diet options. Being aware of the religious practice of the Muslim people, they also started providing prayer rooms for the patients’ convenience [45]. According to the Ministry of Health and Welfare, the policy of medical tourism was to provide supplementary services tailored to the patients and their families. They have also expanded their specialization in languages, such as Arabic, by developing tourism brochures for international medical tourists. The consideration and respect for the Muslim culture in all enterprises that carry out medical tourism is necessary.

## 5. Discussion: The Implications of Medical Tourism between South Korea and the UAE

### 5.1. Medical Tourism as a Medium for Health and Medical Cooperation

Because of the lack of development in the domestic medical industry, the UAE’s government has suffered enormous economic losses in recent years (i.e., an increase in the financial burden due to the high cost of overseas medical treatment). Citizens of the UAE have favored traveling overseas for medical treatment due to the lack of medical facilities and professional staff within their country. To counteract this negative phenomenon, the UAE planned to become an “importer” of medical tourism instead of an “exporter” by modernizing its health system based on technical, regulatory, and human resources. The UAE’s medical tourists prefer to stay in their own country for the stability and comfort of treatment and medical facilities. This could be a reason why neighboring Middle Eastern countries are attracting patients to their home countries. This means that, in the long-term, the UAE needs to establish a strong domestic medical industry by constructing medical institutions.

In response to this need, the UAE established a medical hub in its country, and the strategies implemented to reduce the government’s financial burden have been successful thus far. The UAE has established a large medical complex to attract foreign patients from the Middle East, Africa, Russia, and central Asia. The complex medical center in Dubai was created to attract foreign patients from outside with a product packaged in strong support from the government. Additionally, the Dubai Health Authority (DHA) and Dubai Tourism and Commerce Management are working with hospitals and hospitality providers to offer healthcare packages for medical tourists [45].

The UAE authorities say that medical tourism has increased steadily and will keep increasing in the future. For example, the UAE Medical and Pharmaceutical Reports from 2011 say that “The number of medical tourists increased to 135,000 last year, from 120,000 in 2013, according to Dubai Health Authority (DHA)” [39]. In addition, the DHA stated that “Medical tourism is forecast to increase 15% to Dh1.1bn ($299.5 million) in 2016 from Dh652m ($177.5millio) in 2012. This figure will climb again to Dh2.6 billion ($708 million) in 2012. DHA figures released showed that in addition to the Dh8.5bn ($2.3 billion) spent on healthcare in Dubai in 2012, a further Dh1.5bn ($408 million) was spent outside the emirate, often to pay for treatments or specialist care that could only be found abroad” [44]. In other words, the UAE has been promoting the growth of medical tourism with political support to facilitate the financing.

At the same time, the government has also encountered problems regarding its policies on medical tourism, including the lack of skilled personnel. Therefore, exporting specialized and skilled medical personnel from South Korea to the UAE could be a good opportunity. Medical tourism and the health care industry will play a major role in developing the industry by creating more jobs and fostering a variety of areas for the industry.

In recent years, the relationship between South Korea and the UAE has progressed as a result of medical tourism. Table 6 shows the type of medical cooperation. The two countries have a common interest in health and medical cooperation that resulted in the creation of new policies. Therefore, medical tourism is an opportunity to increase cooperation between the two countries.

The Ministry of Health and Welfare has stated that promoting health services will benefit the national interests of both countries through the exchange of technology, information, and experiences in the medical field, as well as through the exchange of regulations regarding health policies and management [37].

The areas of common interest and avenues for cooperation between businesses highlight the necessity of the two nations’ collaboration. The data show that the common areas of interest in the health and medical sectors should be the starting point for the expansion of healthcare business in the UAE.

Therefore, the medical tourism sector is the recommended method to integrate business sectors of common interest. According to *Tourism, Regional Development, and Public Policy*, “Research on tourism policy has mainly centered on these new strategies for destination development, in particular the difficulties inherent in attempts to orchestrate changes that involve a combination of very different services” [46].

In 2014, South Korea achieved remarkable growth through this cooperation. According to South Korea’s Government Website—News in Cheong Wa Dae—the government is straightforward, as expressed through the strategic partnership between South Korea and the UAE after President Park Geun-hye visited the four countries of the Middle East. The direction of the political partnership was emphasized based on that premise, while also emphasizing the potential for economic cooperation [47].

Under the Abu Dhabi Economic Vision 2030, the UAE promoted new opportunities for both countries, such as information, communication technology, new renewable energy, and healthcare, and should expand cooperation opportunities in promising sectors. [48].

With globalization, the importance of exchanges has become an even greater focus, and this has also made medical tourism a more important high-value-added industry. According to the Ministry of Health, the UAE is trying to foster this high-value industry by strengthening central and local governments’ healthcare services and standards through strategic policies. Opportunities to promote collaboration between the two countries began to arise when the UAE tried to establish a profound medical hub.

The rapid increase in the medical tourism of patients going from the UAE to South Korea, especially through patient transfer arrangements signed with the UAE Health Authorities and the Health Administration of Abu Dhabi, have given the UAE’s government a certain impression of South Korea’s medical technology and their health system’s capabilities. This, in turn, has contributed to the expansion of efforts to establish a partnership in the health and medical sectors.

### 5.2. Medical Tourism as a Mediator for Cultural Exchange

To form a strong relationship with other countries, the relationship must go beyond politics or economic cooperation. Cultural exchange is a premise based on which people of each state can understand each other. As such, there must be an increase in cultural awareness, known as the influence of “soft power.” Hitchcock, King, & Parnell (2018) argued, “Culture is a very important means for understanding their identity as an important element to reflect the look and awareness of people’s lives. Cultural identity is as ‘an on-going process, politically contested and historically unfinished,’ and always mixed, relational, and inventive” [49]. Cultural relativity is a key factor in forming a binding bilateral relationship. Therefore, for South Korea to establish a stronger relationship with the UAE, efforts must be made in terms of cultural exchange.

As described in the previous section, cultural exchange has expanded in recent years. Educational exchange between universities, the establishment of a Korean Cultural Center in the UAE, and the Korea Festival all contribute to the enhancement of cultural exchanges, which can contribute to a stronger relationship between these countries.

The Korean wave, known as Hallyu, is also gaining popularity in the Middle East (including in the UAE) as Middle Eastern people are interested and have access to K-pop and Korean movies and dramas. This, of course, will also generate cultural exchanges as citizens from the UAE become more interested in Korean culture. South Korea has introduced this kind of publicity to promote the tourism industry, such as medical tourism and Hallyu. According to the BBC, “The program has been produced and is set to air on the BBC thanks to a combination of Seoul Metropolitan Government’s efforts to attract tourists from Europe and Southeast Asia, who are considerably fewer in number than those from Japan and China, the K-pop fever that is sweeping the world, and the BBC’s plan to shed light on medical tourism in Korea” [50].

Another way of expanding cultural exchange is through tourism. As the United Nations Environment Programme (UNEP) states on its website, “Traveling brings people into contact with each other and, as tourism has an educational element, it can foster understanding between peoples and cultures and provide cultural exchange between hosts and guests. Because of this, the chances increase for people to develop mutual sympathy and understanding and to reduce their prejudices” [51]. This applies to medical tourism as well.

Besides medical treatment benefits, a combination of traditional tourist attractions, hotels, climate, food, and cultural visits with medical procedures are also key factors that are thought to contribute to growth in the medical tourism market. Table 7 shows the total number of visitors from the UAE and medical tourists since 2009.As Table 7 illustrates, the number of visitors from the UAE has been increasing, and the increase rate is getting higher proportionally to the increase in bilateral relations. Another significant feature is that the proportion of medical tourists is increasing from only 1.9% of the total visitors in 2009 to 25.1% in 2014. Therefore, it is important to utilize medical tourism as a means of cultural exchange and to make UAE nationals more familiar with South Korea’s culture. Promoting medical tourism will be a good way to contribute to South Korea’s cultural exchanges with the UAE, as well as for political and economic cooperation. In the future, the UAE may have the highest number of medical tourists visiting South Korea and the largest number of health and medical service agreements.

## 6. Conclusions: Medical Tourism as a Pathway for Relationship Improvement

The various studies presented in this paper have actively highlighted the importance of medical tourism based on previous research undertaken in this area. Many countries consider tourism a “passport for peace,” whereby they seek to improve relations by promoting tourism. Tourism has become implicated in political action and activism in recent years, with it being seen and used as a tool for political and economic change.

As a result of globalization, international tourism has been growing at a faster pace than at any other point in time. The growth of international tourism is being regarded as a contributor to the development of the economy, social cooperation, politics, and cultural relations. According to the report of the UNWTO (United Nations World Tourism Organization), “Tourism can make a significant contribution to the three pillars of sustainable development economic, social, and environmental” [52]. In other words, tourism is not only a commercial activity but is also significantly related to politics, and, as such, international tourism is interrelated with international politics in many aspects.

Medical tourism is a new phenomenon in tourism and a growing market that nations are working to occupy. As it is international by nature, medical tourism has many implications on international relations. For example, according to “The Crisp Report” (2007), “the author commissioned to look at how UK experience and expertise in health could be used to best effect to help improve health in developing countries, argues that by engaging in country-level agreements, and drawing up Memorandum of Understandings between two countries, international recruitment of health professionals can be done ethically and based on a twinning arrangement of reciprocal movement and benefit” [27].

The medical institutes of South Korea are also competing in this field, and South Korea’s government is also promoting medical tourism. However, South Korea’s promotion is not simply carried out from the commercial perspective. The government is also using medical tourism in foreign policy as a means of establishing more comprehensive cooperation in the health and medical sector. When the Minister of Health and Welfare visited the 65th World Health Assembly in Geneva, Switzerland, he held summit talks with ministers of health from Vietnam, Cambodia, Laos, and Mongolia. In these talks, he said: “we are planning to form a consultation group with directors from the bureau of health of each country to support the countries in the development of their medical services.” Even though these political actions may have commercial intent from the perspective of promoting South Korea’s medical tourism, they also function as a promotion of international cooperation.

Such an understanding has a significant implication on relations with the UAE. Since 2009, South Korea has acknowledged the UAE as its only strategic partner in the Middle Eastern region and has strengthened not only economical exchanges but also political and cultural exchanges. However, in order to establish more effective means of strengthening the relationship between the two countries, a new field where the two countries’ interests meet has to be explored. As such, the promotion of medical tourism from the UAE would be an effective means of supporting cooperation in this field, as has been illustrated in the earlier sections.

What is even more meaningful is that the influence of medical tourism goes beyond influencing the relations with the UAE; it also has a spillover effect onto other Middle Eastern countries. As the Minister of Health and Welfare stated in a speech on 16 December 2013 at the ceremony for the opening of the Global Healthcare Cooperation Center for the Middle East, “In the 1970s and 1980s, Korean construction workers and nurses found dreams in the Middle East. Their hard work and diligence laid the foundation for the partnership between the Arab countries and Korea. Now with great pleasure and delight, do we recently witness that healthcare is emerging on the new horizon of our cooperation” [37].

The development of the health and medical industry will provide many opportunities in establishing cooperation with the UAE market. Moreover, it creates an opportunity to cooperate in the international medical health field by establishing relations with other countries. According to the press release issued by the Ministry of Foreign Affairs, “the Vice Minister and the Under-Secretary exchanged views on matters of mutual concern, including ways to promote bilateral relations, including high-level exchanges; ways to boost economic and development cooperation; ways to increase substantive cooperation regarding the Barakah nuclear power plant, health and medical care, Halal food, public security, and Hallyu (Korean Wave); and cultural and youth exchanges” [13].

South Korea should create an opportunity to improve the shortage of medical specialists in the UAE in order to promote the tourism industry. Additionally, South Korea should also expand UAE citizens’ awareness of its medical technology as they are the target of medical tourism. South Korea must put an emphasis on the strength of its medical services when promoting medical tourism through entering the UAE market and should expand its medical tourism to the entire Middle Eastern region. As Middle Eastern countries have recently been promoting “medical diplomacy,” the Middle East’s patients and doctor trainees will enter and will also begin to have an impact on infrastructure, including transportation, tourism, and accommodation. In conclusion, medical tourism is an important medium in international relations.

The COVID-19 pandemic had a negative impact on the global medical tourism market. Since the outbreak of the pandemic, the global medical tourism market has declined dramatically due to the drastic decrease in medical tourists. Recently, as a form of medical tourism, vaccine tourism has started to become popular in several countries. However, only certain countries and a small number of privileged groups are benefiting from this vaccine tourism. However, medical tourism should contribute to ending the pandemic by supporting the benefit of availing the vaccine to more people.

As a qualitative research method, this study presented the political, economic, and cultural cooperation between the two countries through statistical data and figures. Future research will improve the quality of research through in-depth interviews with experts in bilateral cooperation and exchange. Through quantitative as well as qualitative research, we demonstrate the importance of medical tourism as a mediator in exchanges between the two countries.

The limitations of this study are as follows. First, this study highlights the importance of medical tourism as a mediator and presents examples of political, economic, and cultural exchanges between South Korea and the UAE. However, it should be considered that there may be exceptions between countries as this fact cannot be generalized to all countries. Second, we could not present statistical data related to medical tourism in 2020 and 2021 due to the inactivation of medical tourism in the pandemic era caused by COVID 19. A limitation of this study is that it does not sufficiently reflect the current needs of medical tourists. Future research should focus on the development of medical tourism in the post-corona era.

## Figures and Tables

**Table 1:** Chronology: establishment of diplomatic relations.

Date	Diplomatic Relations
10 December 1971	South Korea’s diplomatic recognition of the UAE
18 June 1980	Diplomatic relations established
3 December 1980	Opening of South Korea’s Embassy in the UAE
6 May 1987	Opening of the UAE’s Embassy in South Korea
3 December 2008	Consulate General opened in Dubai
2009	Strategic Partnership (the Barakah nuclear power plant as a milestone project
2018	Special Strategic Partnership (all areas of cooperation at the political, economic, cultural, and other aspects)

Source: Adapted with permission from Ref. [32]. 2022, Ministry of Foreign Affairs & International Cooperation.

**Table 2:** Major agreements between South Korea and the UAE.

Major Agreements	Agreement Date	Effective Date
Investment Guarantee Agreement	June 2002	June 2004
Double Taxation Agreement	September 2003	March 2005
Aviation Agreement	November 2005	July 2010
Economic, Trade, and Technical Cooperation Agreement	May 2006	April 2008
Military Cooperation Agreement	May 2007	February 2008
Cultural Cooperation Agreement	May 2007	February 2008

Source: Ministry of Foreign Affairs of the Republic of Korea, Outlook on the UAE (2015) and Ministry of Foreign Affairs & International Cooperation of the UAE (2022). On the UAE side, His Highness Sheikh Mohamed bin Zayed Al Nahyan, Crown Prince of Abu Dhabi and Deputy Supreme Commander of the UAE Armed Forces, visited the Republic of Korea in May 2010, March 2012, April 2014, and February 2019. On the Korean side, the most prominent visits were the ones made by former Presi-dent Lee Myung-bak in 2009 and 2011, former President Park Geun-hye in 2014 and 2015, and President Moon Jae-in in 2018.

**Table 3:** The difference in medical costs between South Korea and the US.

Type of Procedure	South Korea	The U.S.
Heart Bypass	$24,000	$144,000
Heart Valve Replacement	$36,000	$170,000
Hip Replacement	$16,450	$43,000
Knee Replacement	$17,800	$50,000
Spinal Fusion	$17,350	$100,000

Source: Adapted with permission from Ref. [34]. 2015, Handley [34].

**Table 4:** Total number of foreign patients by nationality.

Nationality	2012	2013	2014	2015	2016	2017	2018	2019	Average YoY Increase Rate
China	32,503 (20.4%)	56,075 (26.5%)	79,481 (29.8%)	99,059 (33.4%)	127,648 (35.0%)	99,837 (31.0%)	118,310 (31.2%)	289,845 (26.9%)	44.2%
The U.S.	30,582 (19.2%)	32,750 (15.5%)	35,491 (13.3%)	40,986 (13.8%)	48,788 (13.4%)	44,440 (13.8%)	45,213 (11.9%)	138,231 (12.8%)	35.5%
Russia	16,438 (10.3%)	24,026 (11.4%)	31,829 (11.9%)	20,856 (7.0%)	25,533 (7.0%)	24,859 (7.7%)	27,185 (7.2%)	101,385 (9.4%)	49.5%
Japan	19,744 (12.4%)	16,849 (8.0%)	14,336 (5.4%)	18,884 (6.4%)	26,702 (13.4%)	27,283 (8.5%)	42,563 (11.2%)	97,437 (9.0%)	33.0%
Kazakhstan	1633 (1.0%)	2890 (1.4%)	8029 (3.0%)	12,567 (4.2%)	15,010 (4.1%)	12,566 (3.9%)	12,987 (3.4%)	51,946 (4.8%)	88.3%
Mongolia	8,407 (5.3%)	12,034 (5.7%)	12,803 (4.8%)	12,522 (4.2%)	14,798 (4.1%)	13,872 (4.3%)	14,042 (3.7%)	50,555 (4.7%)	45.8%
Vietnam	2231 (1.4%)	2988 (1.4%)	3728 (1.4%)	5316 (1.8%)	8746 (2.4%)	7447 (2.3%)	7532 (2.0%)	31,168 (2.9%)	66.6%
Canada	2756 (1.7%)	2770 (1.3%)	2943 (1.1%)	3206 (1.1%)	4123 (1.1%)	3966 (1.2%)	4098 (1.1%)	9519 (0.9%)	25.2%
The UAE	342 (0.2%)	1151 (0.5%)	2633 (1.0%)	2946 (1.0%)	3562 (1.0%)	3384 (1.1%)	3034 (0.8%)	4089 (0.8%)	59.6%
Uzbekistan	824 (0.5%)	1358 (0.6%)	1904 (0.7%)	2634 (0.9%)	4103 (1.1%)	3253 (1.0%)	3915 (1.0%)	10,475 (1.0%)	52.3%
Total	159,464	211,218	266,501	296,889	364,189	321,574	378,967	497,464	

Source: Adapted with permission from Ref. [14]. 2019, Korea Health Industry Development Institute. YoY: year on year.

**Table 5:** The Middle East: types of medical treatments (2019).

Type	Patients
General Medicine	3077
Dermatology	1041
Oriental Medicine	938
Pediatrics	907
Plastic Surgery	851
Orthopedics	760
Dental Treatment	603
Health Screening, etc.	7151
Total	15,328

Source: Adapted with permission from Ref. [14]. 2019, Korea Health Industry Development Institute [14].

**Table 6:** Types of medical cooperation between South Korea and the UAE.

Area of Common Interest	Cooperation Proposals
Health policies and management	Information exchange
Medical services	Exchange program for delegates and experts
Medical technology and equipment	Exchange of experiences on matters of mutual interest to both countries
E-health	Attending conferences, meetings, and other events
Health and pharmaceutical policy and research	International cooperation
Research and development and the industrialization of biotechnology	Health, medical, and personnel training carried out by joint projects in the pharmaceutical sector
Other areas of common interest determined by the agreement	Promoting access to health services between the two countries

Source: Adapted with permission from Ref. [21]. 2015, The Ministry of Health and Welfare.

**Table 7:** The number of visitors and medical tourists from the UAE.

	2009	2010	2011	2012	2013	2014	2015	2016	2017	2018	2019
Total visitors	887	2433	2958	3866	6098	10,501	9490	10,690	10,990	11,427	13,226
(Increase rate, %)		(174.3)	(21.6)	(30.7)	(57.7)	(72.2)	(−9.6)	(12.6)	(2.8)	(4.0)	(15.7)
Medical tourists	17	54	158	342	1151	2633	2946	3562	3384	3034	4089
(Increase rate, %)		(217.6)	(192.6)	(116.5)	(236.5)	(128.8)	(11.9)	(20.9)	(−5.0)	(−10.3)	(34.8)

Source: Revised from the Korea Tourism Organization and Korean Health Industry Development Institute.

## Data Availability

No new data were created or analyzed in this study. Data sharing is not applicable to this article.
